# Adaptive feature fusion network for machine fault diagnosis with multiple knowledge based graphs

**DOI:** 10.1038/s41598-026-49201-y

**Published:** 2026-04-21

**Authors:** ChangChao Liu, Jinzhe Han, Yue Zhang, Zhizheng Zhang, Hui Gao, Lei Jia

**Affiliations:** 1 School of Control Sciences and Engineering, Shandong University, Jinan, 250061 Shandong China; 2 Institute of Marine Science and Technology, Shandong University, Qingdao, 266237 Shandong China

**Keywords:** Intelligent fault diagnosis, Deep learning, Graph neural networks, Adaptive feature fusion, Graph embedding, Electrical and electronic engineering, Computational science, Information technology

## Abstract

In the field of intelligent fault diagnosis, graph neural networks (GNNs) can create a richer fault feature space by modeling dependencies between sensor signals and embedding them in a structural attribute graph. However, existing GNNs models typically use monitoring signals to directly construct graphical datasets, which suffer from poor representation and edge redundancy. To handle fault diagnosis tasks in noisy environments, the feature representation of graph data and the adaptability of GNNs models need improvement. This paper proposes a hybrid graph neural network based on an adaptive feature fusion network (AFFN-HGNN) for rotating machinery fault diagnosis to address these challenges. First, we introduce an approach to construct multiple knowledge-based graphs, enhancing the exploration of fault information from various perspectives and levels. Next, an adaptive feature fusion network is developed to a) adaptively fuse the time-spatial dependencies from the multiple knowledge-based graphs and hidden correlations between nodes and b) dynamically adjust the fusion weights of each feature layer to improve learning efficiency while minimizing computational overhead. Experimental results on three mechanical systems in different noisy environments show that the proposed method offers improved robustness and accuracy compared to recent GNNs-based fault diagnosis models.

## Introduction

Failures in rotating machinery can cause production delays, equipment damage and even safety accidents, therefore, timely and accurate fault diagnosis is essential to ensure stable equipment operation^1^. Traditional methods, such as physical models, statistical approaches and expert systems, rely heavily on prior knowledge and expert experience^2^. However, as equipment becomes larger and more complex, these methods suffer from poor adaptability and low efficiency^3^. It is essential to research advanced fault diagnosis techniques for modern manufacturing equipment to ensure stable and safe operation.

In recent years, deep learning-based fault diagnosis methods have enabled end-to-end intelligent fault detection by performing multi-level automatic feature extraction and efficient fault pattern recognition on sensor data^4–6^. CNNs are particularly effective at extracting local features, allowing them to quickly identify healthy states and map fault patterns^7^. Xia et al.^8^ applied CNNs to extract and fuse features from multi-sensor vibration signals. Wen et al.^9^ used CNNs to extract features from grayscale images of vibration signals for fault classification. Li et al.^10^ employed multiple CNNs to directly extract fault features from one-dimensional vibration signals and trained a Gaussian Mixture Bayesian model, achieving highly accurate end-to-end fault diagnosis for rotating machinery. However, when condition monitoring signals are affected by noise, local feature extraction using CNNs may fail to accurately capture fault information^11^. As a result, researchers are increasingly focusing on extracting long-term dependencies from sensor sequences to enhance the ability of model to resist interference from local noise.

RNNs and their variants, such as Long-Short Term Memory (LSTM) and Gated Recurrent Units (GRU), use a recurrence mechanism to learn long-term dependencies in time-series data^12,13^. Ravikumar et al.^14^ developed a fault diagnosis method for gearboxes based on stacked LSTM networks. Shi et al.^15^ used time-frequency domain features from gearbox monitoring signals as input to the LSTM, achieving accurate fault detection. Belagoune et al.^16^ demonstrated the potential of LSTM for large-scale fault area identification and fault location prediction in power systems. Cabrera et al.^17^ optimized LSTM hyperparameters with Bayesian networks to improve fault diagnosis accuracy for compression pumps. These applications showcase the strong ability of RNNs and their variants to extract and recognize fault features. However, the above deep learning models mainly focus on extracting fault features from the monitoring data itself. They are unable to embed the potential relationships between sample elements into the extracted spatial features, making it difficult to effectively handle varying operating conditions and to detect mixed fault features^18–20^.

GNN is designed to process graph-structured data. The edges of the graph are used to capture the dependencies between neighboring nodes, creating edge features that enrich the feature space of the original data^18^. Researchers have developed various GNN variants, such as Graph Convolution Networks (GCN)^19,21^, Graph Attention Networks (GAT)^20,22^, Graph Recurrent Neural Networks (GRNN)^23^, and Graph Autoencoders(GAE)^24^, which have been applied to intelligent fault diagnosis. Zhang et al.^25^ were the first to apply GCN to rolling bearing fault diagnosis. They converted collected acoustic signals into graphs and used GCN to model these graphs for fault detection. Wang et al.^26^ proposed a GCN-based fault classification method for multiple microscopic images, which reduced the computational complexity of processing large image datasets. GCN is effective in information fusion because it aggregates neighborhood information. However, most GCN methods focus on constructing graph-structured data and optimizing models, but not fully explore how feature learning can be improved by different types of message aggregation^27^.

The Graph Attention Network, which uses an attention mechanism, can dynamically focus on important information from neighbouring nodes, enhancing its ability to learn key features in the graph structure^20^. Yang et al.^28^ proposed a semi-supervised fault feature extraction and classification method for rotating machinery based on the GAT model. Abudurexiti et al.^29^ tackled the issue of underutilized sample relationships in fault diagnosis by introducing a feature representation method that combines an improved GAT, which boosted model performance.

However, since there is no clear dependency between vibration signal samples, directly using them to construct graph data does not provide effective feature information. To address the problem of insufficient fault information extraction in high-frequency vibration signals under strong noise conditions, this paper proposes a multiple knowledge-based graph construction method that helps reveal the inherent relationships between different elements of the data from multiple levels. In addition, a fault diagnosis model based on adaptive feature fusion in graph neural networks is proposed, which adaptively fusion the output of different feature extraction layers to enable more comprehensive fault information extraction.

The main contributions of this work are as follows:

$\bullet$ Multiple knowledge-based graphs are constructed using similarity measures to enhance the fault information representation space of rotating machinery from various perspectives. The KNN algorithm measures the distance between nodes, helping capture the local structure of samples. Cosine similarity strengthens the connections between nodes with the same fault patterns by measuring similarity in the feature space. Temporal structure graphs utilize time dependencies in the data to reveal long-term trends between samples and capture the dynamic features of fault evolution.

$\bullet$ A hybrid graph neural network (HGNN) model is proposed. First, GAT is used to learn the feature weight relations during sample message passing, which captures local feature information. Then, multi-layer GCN is applied for higher-order message passing to further reveal deeper structural information. By combining the strengths of GAT and GCN, the hybrid model learns features from different perspectives, enhancing its ability to represent data and improve fault pattern recognition in complex noisy environments.

$\bullet$ A new adaptive feature fusion network is constructed to adjust the fusion weights of the GAT and GCN layers.We designed a loss function integrating entropy loss and similarity loss to measure the diversity and complementarity of graph features. This enables the model to choose the optimum feature learning strategy under different fault modes and data distributions.

## Theoretical background

GNN is a neural network architecture designed for learning graph-structured data. Its core idea is to iteratively update node representations, combining the representations of neighboring nodes with the node itself. The graph structure is represented as ${G=(V,E)}$: *G* denotes the graph, *V* represents the set of nodes, and *E* represents the set of edges. In GNN-based fault diagnosis, each sample is treated as a graph node, with correlations between them represented as edges.

### GCN

GCN is a specialised type of CNN designed for graph-structured data. The graph convolution operation in each layer aggregates information from neighbouring nodes to update the features of the node, ultimately forming a global representation for each node^19^. In the GNN model, the relationships between nodes are represented by the adjacency matrix $A\in \mathbb {R}^{N*N}$. Let $H^k\in \mathbb {R}^{N*F^k}$ represent the node feature matrix at the $k$-th layer, $N$ be the number of nodes, and $F^k$ be the feature dimension at the $k$-th layer. The message passing mechanism of graph convolution is represented as:

$$\begin{aligned} H^{k+1} =\sigma (\hat{A}H^kW^k) \end{aligned}$$

where $\sigma$ is the activation function, and $W^k$ is the weight matrix at the $k$-th layer with shape $F^k \times F^{k+1}$. $\hat{A}=D^{-1\setminus 2}(A+I)D^{-1\setminus 2}$, which represents the normalized adjacency matrix, while $D\in \mathbb {R}^{N*N}$ is the degree matrix of the graph, which is a diagonal matrix with diagonal elements $D_{ii}=\sum _jA_{ij}$, and $I$ is the unit matrix.

For each node $i$ of the $k$-th layer, the feature update process is given by:

$$\begin{aligned} \textbf{h}_i^{(k+1)} = \sigma \left( \sum _{j \in \mathcal {N}(i)} \hat{\textbf{A}}_{ij} \textbf{h}_j^{(k)} \textbf{W}^{(k)} \right) \end{aligned}$$

where $\textbf{h}_i^{(k)}$ is the feature vector of node $i$ at layer $k$, $\mathcal {N}(i)$ is the set of neighbors of node $i$, which includes all adjacent nodes to node $i$, $\hat{\textbf{A}}_{ij}$ is the normalized adjacency matrix element, representing the relationship strength (weight of the edge) between node $i$ and node $j$, $\textbf{W}^{(k)}$ is the weight matrix at layer $k$.

### GAT

GAT enhances the learning ability of graph data by introducing an attention mechanism^20^. At its core, the attention mechanism dynamically assigns weights to different neighbouring nodes for each node to optimise the transmission of information between nodes^11^. This not only improves the adaptability of the model to the neighbourhood importance of each node, but also improves the overall interpretability of the model.

In GAT, the attention coefficient $\alpha _{ij}$ between nodes $i$ and $j$ is computed using a self-attention mechanism:

$$\begin{aligned} \alpha _{ij} = \frac{\exp \left( \text {LeakyReLU} \left( \textbf{a}^\top [\textbf{W} \textbf{h}_i \parallel \textbf{W} \textbf{h}_j] \right) \right) }{\sum _{k \in \mathcal {N}(i)} \exp \left( \text {LeakyReLU} \left( \textbf{a}^\top [\textbf{W} \textbf{h}_i \parallel \textbf{W} \textbf{h}_k] \right) \right) } \end{aligned}$$

where $\textbf{h}_i$ and $\textbf{h}_j$ are the feature vectors of node $i$ and node $j$ at the input layer, $\textbf{W}$ is the learnable weight matrix used to map input features, $\textbf{a}$ is the learnable attention vector used to compute the attention coefficient, $\parallel$ denotes the concatenation of two vectors, $\mathcal {N}(i)$ is the set of neighbors of node $i$, $\text {LeakyReLU}$ is an activation function used to introduce non-linearity.

The feature of node $i$ can be updated by aggregating the features of the neighboring nodes $j$:

$$\begin{aligned} \textbf{h}_i^{(l+1)} = \sigma \left( \sum _{j \in \mathcal {N}(i)} \alpha _{ij} \textbf{W} \textbf{h}_j^{(l)} \right) \end{aligned}$$

where $\alpha _{ij}$ is the attention coefficient between nodes $i$ and $j$, $\sigma$ is an activation function. Thus, each neighboring node’s feature $\textbf{h}_j^{(l)}$ is weighted by $\alpha _{ij}$, which represents the strength of the connection between node $i$ and node $j$. The aggregated features are then transformed by the weight matrix $\textbf{W}$ and updated for node $i$ in the next layer.

## The proposed approach

**Fig. 1:**
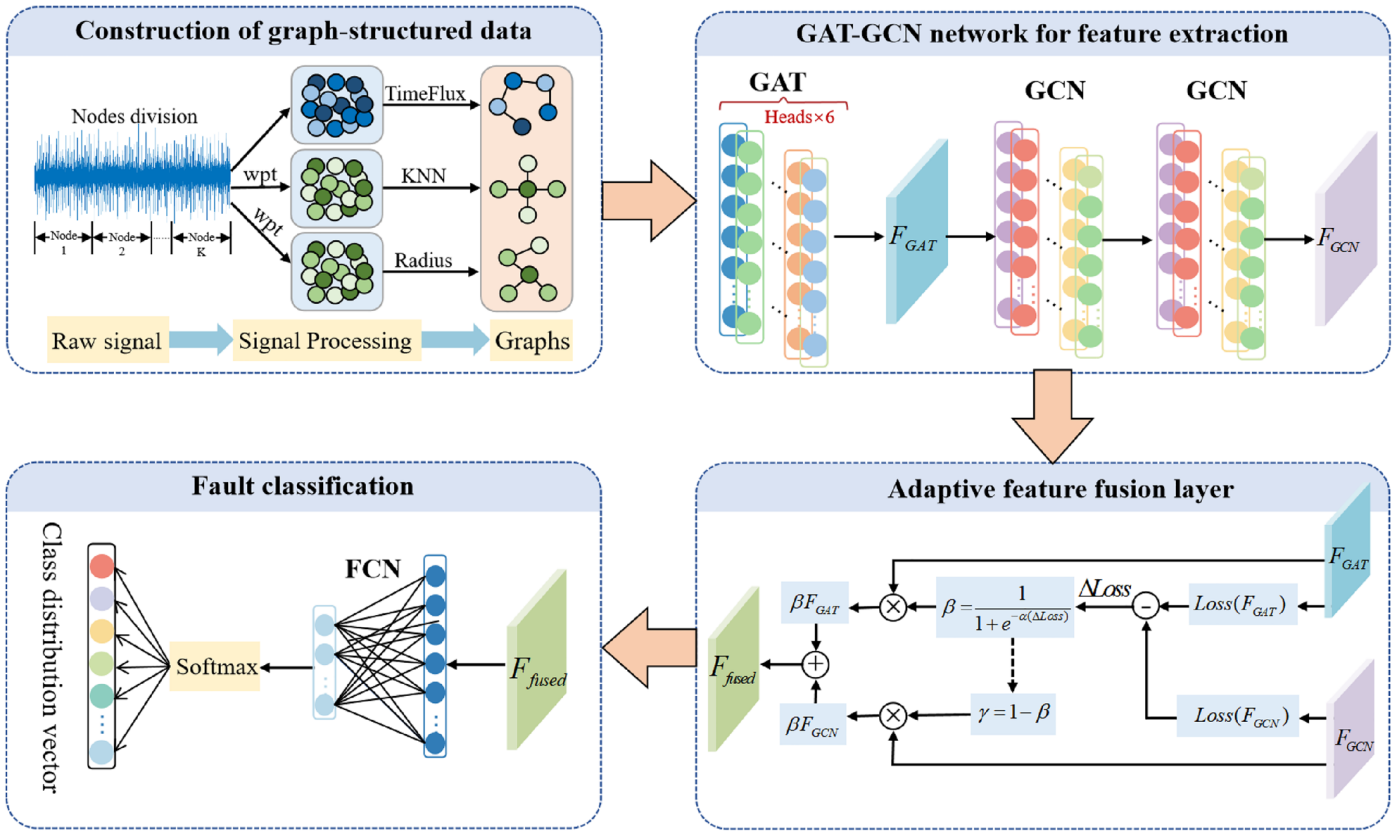
The flow chart of the proposed fault diagnosis approach.

In this section, the process of the novel fault diagnosis approach based on a hybrid Graph Neural Network with Adaptive Feature Fusion Network (AFFN-HGNN) framework and the specific architecture of the AFFN-HGNN framework are introduced. The flow chart of the fault diagnosis approach is shown in Fig. 1. The specific steps are as follows:

$Step$ 1: First, in the data pre-processing stage, multiple knowledge-based graphs are constructed by the sensor data analysis, which represent the structured information of the original data from different perspectives. The graph-structured data will be fed into the GNN network.

$Step$ 2: A hybrid Graph Neural Network is constructed for feature representation learning, which consists of one GAT layer and two GCN layers. The first GAT layer learns the local relationships between nodes and performs feature-weighted aggregation to produce an output known as $F_{GAT}$. The GCN layers capture higher-order graph structure information through multiple convolutions and output $F_{GCN}$.

$Step$ 3: The features learned by the GAT and GCN layers are then fed into AFFN for fusion. AFFN dynamically calculates the fusion factor using an adaptive learning function to improve the capability and adaptability of the model for different fault modes.

$Step$ 4: Finally, the fused features $F_{fused}$ are fed into the fully connected layers for fault classification. The test set is input to the trained model for test of fault diagnosis accuracy, and output the diagnostic results.

### Multiple knowledge-based graphs

**Fig. 2:**
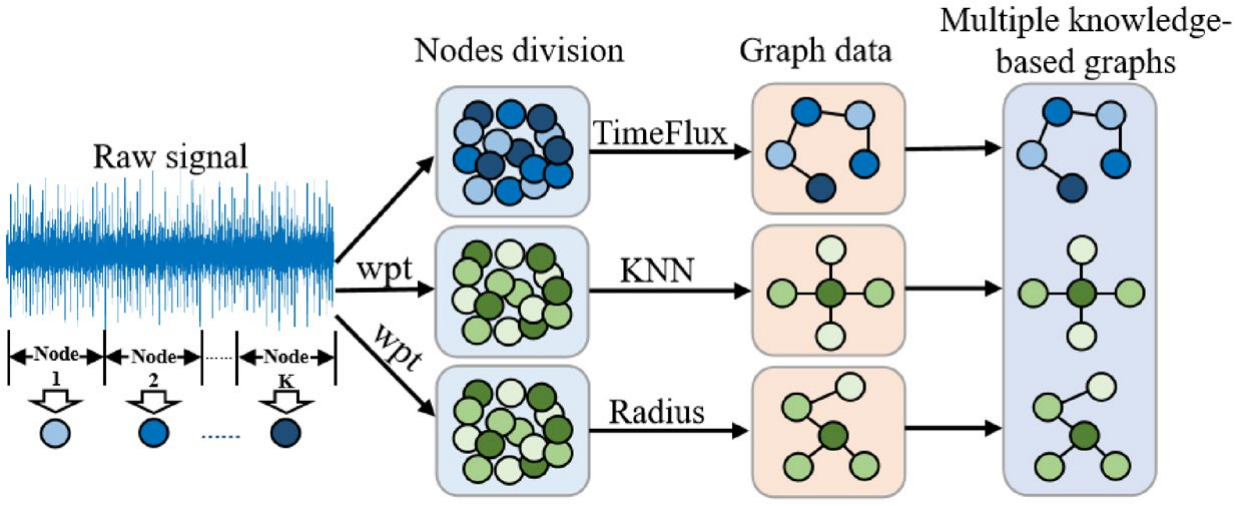
Construction of multiple knowledge-based graphs.

As shown in Fig. 2, This section proposes a novel strategy for constructing multiple knowledge-based graphs with vibration monitoring signals, which enhances data channels to reveal diverse characteristics and intrinsic relationships, thereby improving fault feature representability and robustness in high-noise environments.

This method involves two key steps: dataset processing and constructing graph-structured data. In the data preprocessing step, raw vibration signals are split into fixed-size sub-samples, with each sub-sample acting as a node in the graph^30^. Next, three data analysis techniques are applied to build multiple knowledge-based graphs.

#### Data preprocessing

Sample segmentation: To ensure the independence of each sub-sample and to reduce information redundancy, the normalised high frequency vibration signal is divided into non-overlapping sub-samples, each containing 2048 data points. The resulting subsamples are represented as:

$$\begin{aligned} S = [(x_1,y_1), (x_2,y_2), \dots , (x_k,y_k) ],k=\lceil \frac{l}{d}\rceil \end{aligned}$$

where the obtained sample set is denoted by $S$, a subsample by $x$, the sample label by $y$. The number of sub-samples is denoted as $k$, the sample length is denoted as $l$. The segment size is set as $d$, and in this study $d$ is set to 2048.

**Fig. 3:**
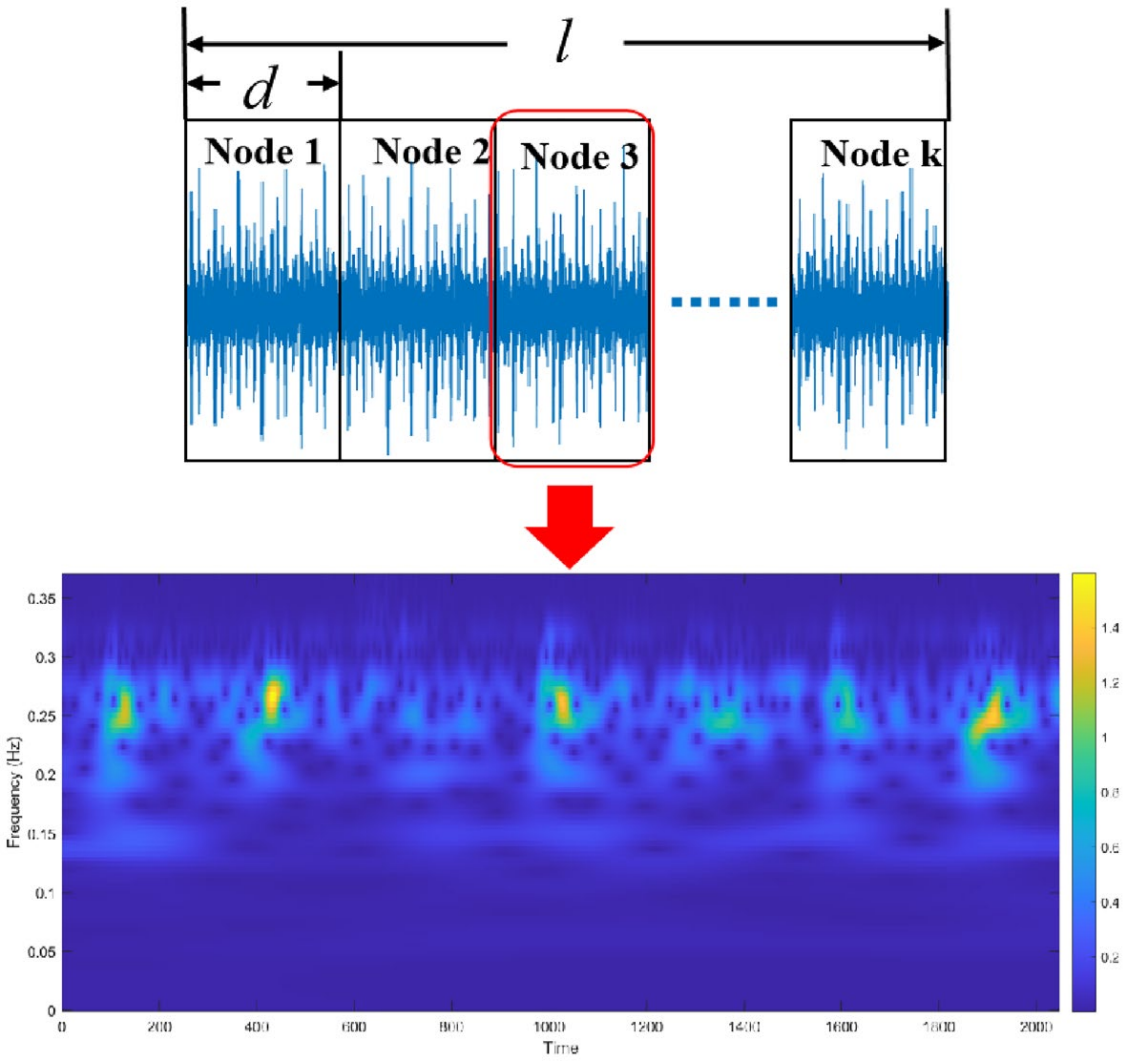
Scheme of sample segmentation and wavelet packet transform.

Wavelet packet transform(wpt)^31^: In this section, Daubechies wavelets are used as the wavelet basis, with the number of wavelet decomposition levels set to 3. The boundary handling mode is set to symmetric to reduce the impact of edge distortion. Through wavelet packet transformation, the original vibration signal is decomposed into multiple frequency components, each represented by the corresponding wavelet packet node^32^. The node data is then sorted and concatenated in order of frequency, from low to high, to construct a sub-sample set. The data splitting and wavelet packet transformation process is shown in Fig. 3.

#### Construction of graph-structured data

In this study, each sub-sample is treated as a node in a graph, and the KNN algorithm, cosine similarity, and temporal relationships are used to establish the adjacency relations between nodes, revealing the data characteristics and their intrinsic connections from multiple perspectives.

1) Graph-structured data based on KNN

The KNN algorithm connects each data point to its $k$ nearest neighbors based on a distance measure, constructing the graph structure^18^. KNN identifies clusters of samples under different fault modes, helping to uncover potential fault features. In this graph structure, the neighboring nodes of node $x_i$ are represented as:

$$\begin{aligned} Ne(x_i) = KNN(k', x_i, \Psi ) \end{aligned}$$

where $k'$ represents the number of nearest neighbors, set to 5 in this experiment as it was found to be the optimal value through a grid search optimizing validation accuracy. and $\Psi = [x_1, x_2, \dots , x_m]$ is the sample set consisting of $m$ samples. The neighbors of $x_i$ are denoted as $Ne(x_i)$. The edge weights between nodes are determined by the Gaussian kernel function:

$$\begin{aligned} a_{i,j} = \exp \left( -\frac{\Vert x_i - x_j\Vert ^2}{2 \xi ^2} \right) , \quad x_j \in Ne(x_i) \end{aligned}$$

where $a_{i,j}$ is the weight of the edge between node $x_i$ and its neighbor $x_j$, and $\xi$ is the bandwidth of the Gaussian kernel.

**Fig. 4:**
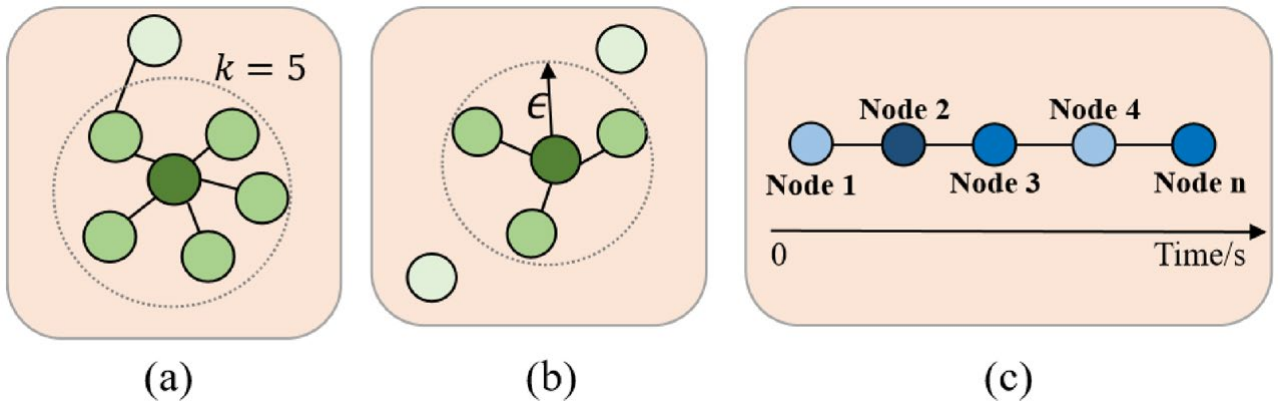
The constructing of the knowledge-based graphs, **a** KNNs graphs; **b** Cosine similarity graphs; **c** Time-sequence graphs.

Figure 4a illustrates a schematic diagram of the KNN algorithm constructing the graph data. The obtained graph data subset $S^K$ is represented as:

$$\begin{aligned} S^K = \bigcup _{i=1}^{m} (x_i, y_i, E_i^K, A_i^K) \end{aligned}$$

where $x_i$ represents the features of the nodes, $y_i$ denotes the node labels, $E_i^K$ is the index of the edges, and $A_i^K$ is the weight of the edges.

2) Graph-structured data based on cosine similarity

Cosine similarity is used to quantify the similarity between node features. Similar feature spaces may correspond to the same fault patterns, helping to establish more accurate feature mapping relationships^33^. In this section, a threshold distance is defined to determine whether an edge should be established between two nodes. The neighboring nodes of node $x_i$ can be obtained using the following formula:

$$\begin{aligned} Ne\left( x_{i}\right) =\epsilon \Theta -radius\left( x_{i},\Psi \right) ,{\textbf {if:}}\epsilon \Theta -radius\left( x_{i},\Psi \right) >\epsilon \end{aligned}$$

where $\Theta -radius(.)$ represents the cosine similarity between node $x_i$ and each node in the set, and $\epsilon$ is the threshold, set to 0 in this paper. This setting, determined via grid search, allows the model to consider all potential positive correlations, with the subsequent graph neural network layers (e.g., GAT) adaptively learning the importance of these edges during training. Figure 4b shows a schematic diagram of constructing graph data based on cosine similarity. The constructed graph data subset $S^C$ is represented as follows:

$$\begin{aligned} S^C = \bigcup _{i=1}^{m} (x_i, y_i, E_i^C, A_i^C) \end{aligned}$$

where $x_i$ represents the features of the nodes, $y_i$ denotes the node labels, $E_i^C$ is the index of the edges, and $A_i^C$ is the weight of the edges.

3) Graph-structured data based on time-sequence

The temporal construction method forms a graph structure by connecting time-adjacent data points. During the sampling process of the original vibration signals, there are temporal dependencies between data points, and the evolution of the temporal graph can effectively reveal the fault’s changing trends. The weight of the edges between neighboring nodes is represented by:

$$\begin{aligned} a_{i,j} = \exp \left( -\frac{\Vert x_i - x_j\Vert ^2}{2 \xi ^2} \right) , \quad x_j \in Ne(x_i) \end{aligned}$$

Figure 4c shows a schematic of constructing temporal graph data. The constructed graph data subset $S^T$ is represented as:

$$\begin{aligned} S^T =\bigcup _{i=1}^{m} (x_i, y_i, E_i^T, A_i^T) \end{aligned}$$

where $x_i$ represents the features of the nodes, $y_i$ denotes the node labels, $E_i^T$ is the index of the edges, and $A_i^T$ is the weight of the edges.

Finally, the graph-structured data subsets $S^K$, $S^C$, and $S^T$ constructed using the three strategies are merged to form the final graph dataset $S$, which is represented by the following equation:

$$\begin{aligned} S = S^K + S^C + S^T \end{aligned}$$

By combining the KNN algorithm, cosine similarity, and temporal construction methods, the graph dataset captures the relationships between samples from multiple dimensions. This multi-perspective representation not only provides a richer feature space for useful information but also helps the fault diagnosis model better uncover the fault characteristics of mechanical equipment, thereby improving diagnostic performance.

The design of the three distinct graph constructions is grounded in complementary principles to comprehensively characterize the fault data.

The **KNN-based graph** is built upon the manifold hypothesis, which posits that high-dimensional data often reside on a low-dimensional manifold. By connecting each node to its k-nearest neighbors, this graph aims to preserve the local geometric structure of the data in the feature space, which is crucial for clustering samples with similar fault patterns and enhancing the model’s discriminative power.

The **cosine-similarity-based graph** operates on the principle that samples sharing the same fault mode exhibit high directional similarity in the high-dimensional feature space, even if their magnitudes differ. This graph strengthens connections between semantically similar nodes, facilitating the model’s ability to learn robust feature representations that are invariant to certain amplitude variations.

The **time-sequence-based graph** is motivated by the temporal dynamics inherent in rotating machinery vibration signals. Fault evolution often follows a chronological pattern, and this graph explicitly captures these inherent temporal dependencies, allowing the model to recognize the progression of faults and trends that are not apparent in the feature or similarity space alone.

The fusion of these three graphs, therefore, provides a more holistic and robust representation by integrating local structural, global semantic, and dynamic temporal information.

### Fault diagnosis model based on AFFN-HGNN

#### Feature extraction modules

**Fig. 5:**
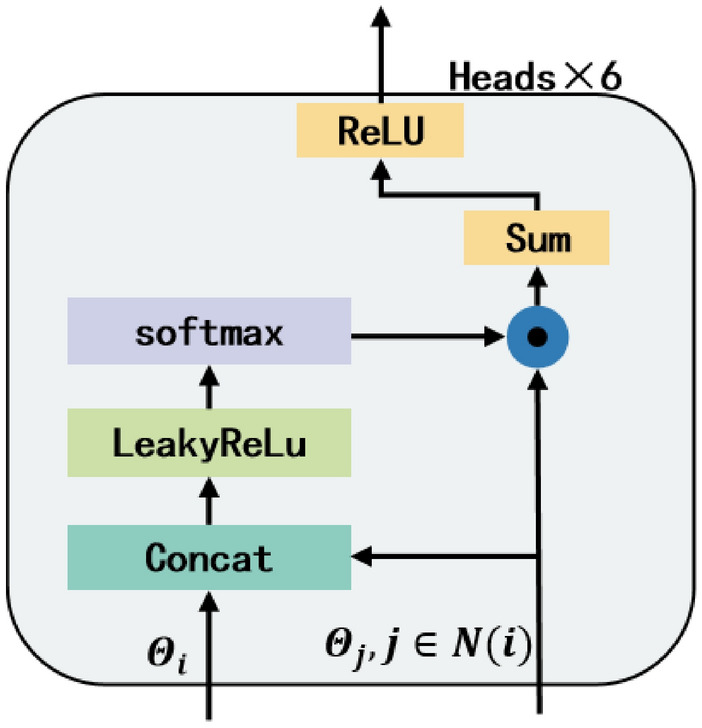
Multihead graph attention convolutional layer.

In the AFFN-HGNN model proposed in this paper, the first layer of the GAT uses a multi-head attention mechanism. The structure of the multi-head graph attention convolution layer is shown in Fig. 5. The process first concatenates the node feature $\theta _i$ with its neighboring node features $\theta _j$, and inputs them into a Softmax layer to compute the attention coefficient for that node. Then, the attention coefficient is multiplied by the neighboring node features through a dot product operation to obtain the updated node features.

Assume the input to the GAT layer is a set of node features $\theta =\left\{ \vec {\theta }_{1},\vec {\theta }_{2},\ldots ,\vec {\theta }_{n}\right\}$. Based on the GAT message-passing mechanism described in Subsection 2.2, the output of the network is $\theta ^{\prime }=\left\{ \vec {\theta }_{1}^{\prime },\vec {\theta }_{2}^{\prime },\ldots ,\vec {\theta }_{n}^{\prime }\right\} ,\vec {\theta }_{i}^{\prime }\in \mathbb {R}^{F^{\prime }}$, where ${F^{\prime }}$ represents the dimension of the new node features. The multi-head attention mechanism is defined as follows:

$$\begin{aligned} Maltihead(\vec {\theta }_{i}^{\prime })=\frac{1}{H}\sum _{h=1}^H (\vec {\theta }_i^{\prime ,h}) \end{aligned}$$

where $H$ denotes the number of attention heads, and $H$is set as 6. In this way, the model can learn the features of the nodes from multiple perspectives, thus improving performance and stability.

In the AFFN-HGNN model, the GAT layer is followed by two layers of GCN to further capture dependency information from broader neighborhoods. The hybrid architecture of one GAT layer followed by two GCN layers embodies a deliberate refine-then-propagate design philosophy. The first GAT layer serves as a feature refinement stage, leveraging attention mechanisms to weight and filter the most informative local relationships from our multi-perspective graph inputs. This creates a contextually enriched feature representation.

Subsequently, the two GCN layers function as a feature propagation module, efficiently aggregating and disseminating these refined features across the graph structure. This configuration strategically places the computationally intensive attention operation at the entry point for critical feature selection, while utilizing the efficiency of graph convolutions for broader feature integration. The two-layer depth represents an optimal balance, enabling sufficient receptive field coverage without introducing excessive complexity or over-smoothing.

The second GCN layer processes the node representations, integrating features from direct neighbors and adjusting their importance using attention weights. The third GCN layer then passes this neighborhood information, enhanced by the attention mechanism, strengthening the ability to capture the global graph structure. According to the principles of GCN described in Section 2.1, the graph structure composed of nodes serves as the model input. The final output of the node features can be represented by the following equation:

$$\begin{aligned} G^{(ouput)}=\Gamma \left( A,G,\Theta \right) \oplus \tau \end{aligned}$$

where $\Theta$ represents the learnable parameters of the network, $\tau$ denotes the fusion weights, $\oplus$ stands for the adaptive fusion operation, $G$ represents the input node features, $\Gamma$ refers to the multi-layer graph convolution operation, and $G^{(ouput)}$ is the final extracted features.

#### Adaptive feature fusion network

This paper proposes a new adaptive fusion network structure to enhance the model’s learning ability by connecting different feature extraction layers. The first GAT layer effectively assigns weights to information passing between nodes, enabling fast convergence when local features are prominent. The third GCN layer gradually aggregates global information from neighboring nodes through multiple layers, uncovering more hidden higher-order features. The output of AFFN is represented as:

$$\begin{aligned} F_{fuse} = \beta F_{GAT} + \gamma F_{GCN} \end{aligned}$$

where $F_{GAT}$ and $F_{GCN}$ represent the output features of the first (GAT layer) and third (GCN layer) respectively. $\beta$ and $\gamma$ are the adaptive fusion weights of AFFN. We proposes a novel adaptive learning function that uses multiple feature evaluation methods to adjust the values of $\beta$ and $\gamma$:

$$\begin{aligned} & \beta = \frac{1}{1+e^{-\alpha (\Delta Loss)}} \\ & \gamma = 1 - \beta \\ & \Delta Loss = Loss(F_{GAT}) - Loss(F_{GCN}) \end{aligned}$$

In this formulation, the hyperparameter $\alpha$ acts as a scaling factor that modulates the sensitivity of the fusion weight $\beta$ to the loss difference $\Delta Loss$. The function $\beta = 1 / (1 + \exp (-\alpha \cdot \Delta Loss))$ is a sigmoid function. We set $\alpha = 1$ in this study. This choice is empirically sound and standard for such gating mechanisms, as it provides a smooth and balanced transition for $\beta$ based on $\Delta Loss$ without introducing additional nonlinearities that require complex interpretation. A detailed analysis of the impact of $\alpha$ and the robustness of the model to its variation is provided in Section 4.7.

The loss function $Loss(.)$ measures the feature learning effectiveness of different hierarchical networks and consists of two parts: entropy loss $L_{entropy}(.)$ and similarity loss $L_{simil}(.)$.

Assuming the feature matrix is $F \in \mathbb {R}^{N \times d}$, where $N$ represents the number of nodes and $d$ the feature dimension of each node. Smaller entropy values indicate more concentrated feature distributions. This encourages the model to learn more discriminative features by penalizing smaller entropy values. $L_{entropy}(.)$ is calculated as follows:

$$\begin{aligned} & p(F_i) = \frac{\exp (F_i)}{\sum _{j=1}^{N} \exp (F_j)} \\ & L_{\text {entropy}}(F) = -\sum _{i=1}^{N} p(F_i) \log (p(F_i)) \end{aligned}$$

The similarity loss is calculated by measuring the similarity between features. When the similarity between elements of the features is high, it indicates insufficient feature representation ability. Therefore, the similarity loss is used to encourage diversity among features, prompting the model to learn more diverse features from multiple levels. $L_{simil}(.)$ is calculated as follows:

$$\begin{aligned} L_{\text {simil}}(F) = 1 - \frac{1}{N^2} \sum _{i,j=1}^{N} \text {cos}\_\text {sim}(F_i, F_j) \end{aligned}$$

By combining the above two losses, the final comprehensive loss function is obtained:

$$\begin{aligned} Loss(F) = L_{\text {entropy}}(F) + L_{\text {simil}}(F) \end{aligned}$$

The adaptive learning function dynamically adjusts the fusion weights, allowing the model to automatically select the most suitable feature extraction path. The GAT layer efficiently captures local information, helping to achieve fast model convergence when degenerate features are significant. For complex data where degenerate features are less obvious, the GCN layer aggregates higher-order neighbour information through multi-layer convolution, allowing to uncover hidden fault features and further improve diagnostic accuracy.

## Experiments

To evaluate the performance of the proposed AFFN-HGNN fault diagnosis model, we perform case studies on three benchmark datasets:1)The Paderborn dataset, which is built by collecting data from bearings in different states on a test rig. The fault types include outer ring faults and inner ring faults;2)The CWRU data set, which is collected from a test rig set up in the laboratory at Case Western Reserve University. Failures are simulated on the rolling element, inner race and outer race;3)The SEU gear data set, which contains five types of failure: chipped tooth, missing tooth, root failure, surface failure and healthy operating condition.

### The model parameters

The objective function used in this method is the cross-entropy loss function, expressed as follows:

$$\begin{aligned} L_{CE}(y, \hat{y}) = -\sum _{j=1}^C y_j \log (\hat{y}_j) \end{aligned}$$

where $C$ represents the number of classes, $y_j$ is the true label (one-hot encoded) for class $j$, and $\hat{y}_j$ is the predicted probability for class $j$.

The optimizer applied in this paper is the AdamW (Adam Weight Decay) optimizer. By separating weight decay from gradient updates, weight decay is treated in a more standardized form, closer to traditional L2 regularization. By decoupling weight decay and gradient updates, AdamW allows weight decay to be adjusted independently of the adaptive gradient update process, thereby providing more effective regularization and helping to improve the model’s generalization ability.

The hyperparameters of the proposed model, including the architectural parameters (e.g., layer dimensions), optimization parameters (e.g., learning rate, batch size), and graph construction parameters (e.g., KNN’s $k'$, cosine similarity’s $\epsilon$), were tuned via a grid search strategy to maximize performance on the validation sets. The final configuration of key parameters is summarized in Table 1. Through iterative training, the main parameters of the AFFN-HGNN model are shown in Table1, where *C* represents the number of fault classes, ${G^{(0)}}$ the dimension of the input data.

**Table 1:** Default hyperparameters for the proposed method.

Name	Parameter	Value
1st GAT Layer	GATConv1	$G^{(0)} \times 256$
2nd GCN Layer GCN	GCNConv2	1536 $\times$1536
3rd GCN Layer	GCNConv2	1536 $\times$1536
Batch Normalization Layer	BatchNorm	1536
1st Fully Connected Layer	fc1	1536 $\times$512
2nd Fully Connected Layer	fc2	512 $\times$C
Dropout	dropout	0.2
Batch Size	batch_size	64
Learning Rate	lr	0.01
Weight Decay	weight_decay	5e-4
Initial Fusion Weights	$\beta , \gamma$	0.5

### Comparison of fault diagnosis performance

#### Experimental verification with the Paderborn dataset

1. Experimental setup

In the Paderborn dataset, based on the damage location, the health states can be categorized into healthy, inner race fault, and outer race fault, with labels 0, 1, and 2, respectively, as shown in Table 2. The high-frequency vibration signals are segmented into 1024 sub-samples, each containing 2048 data points. Each sub-sample is treated as a node in the graph, and every 8 sub-samples are used to construct a graph. The data is divided into training and testing sets in an 8:2 ratio for model training and testing.

**Table 2:** Bearing data for training and testing.

Health condition	Healthy	Inner race fault	Outer race fault
Labels	0	1	2
Bearings	K001	K104	KA04
	K002	K114	KA15
	K003	K116	KA16
	K004	K117	KA22
	K005	K118	KA30

2) Experimental results and analyses

**Fig. 6:**
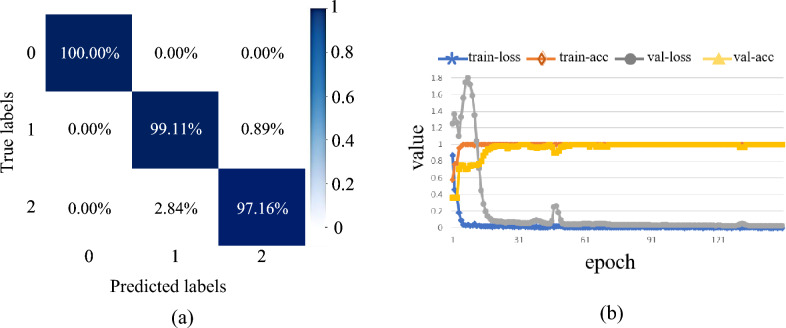
**a** Confusion matrix and **b** learning curve on Paderborn dataset.

The test results are shown in Fig. 6a. The vertical axis of the confusion matrix represents the true labels of the data samples, and the horizontal axis represents the predicted labels. From the figure, we can see that 0.89% of inner race faults (label 1) were incorrectly predicted as outer race faults, and 2.84% of outer race faults (label 2) were incorrectly predicted as inner race faults.

Figure 6b shows the learning curve. As the epoch increases, both the training and testing accuracy curves rise steadily and approach 1, indicating a rapid improvement in accuracy. The loss curves for both the training and testing sets converge quickly and gradually decrease towards 0, indicating that the model did not overfit. The experimental results show that on the Paderborn dataset, the model tends to confuse inner and outer race fault, leading to classification errors. However, it achieved 100% accuracy in predicting the healthy state and accurately distinguished between healthy and faulty states.

#### Experimental verification with the CWRU dataset

1. Experimental setup

In this experiment, a portion of the CWRU dataset with a 48 kHz sampling frequency was used. This dataset includes four bearing health condition categories: healthy, inner race fault, outer race fault, and rolling element fault. Following the data processing method described in section 3.1, the high frequency vibration signal was segmented into 1024 sub-samples, each containing 2048 data points. Each sub-sample was treated as a node in the graph, and a graph was constructed for every 8 sub-samples. The data was split into a training set and a testing set at an 8:2 ratio for model training and testing.

2) Experimental results and analyses

**Fig. 7:**
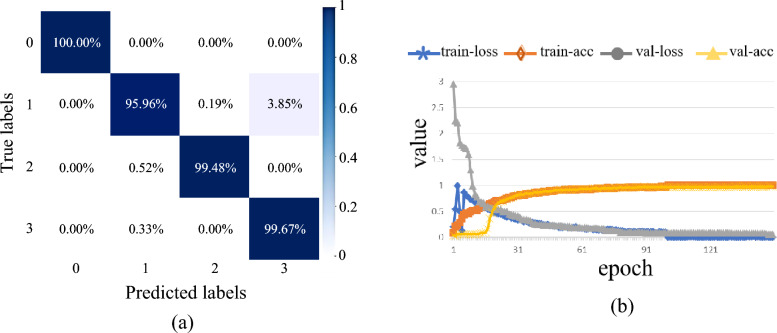
**a** Confusion matrix and **b** learning curve on CWRU dataset.

The test results are shown in Fig. 7a. where the vertical coordinates of the graph indicate the true labels of the samples and the horizontal coordinates indicate the classification results. As can be seen from the graph, 0.19% of the inner ring faults (label 1) were misclassified as outer ring faults and 3.85% of the inner ring faults (label 1) were misclassified as rolling body faults. There were 0.52% of outer ring faults (label 2) incorrectly diagnosed as inner ring faults and 0.33% of rolling body faults (label 3) incorrectly diagnosed as inner ring faults. Figure 7b shows the learning curve. Both the training and testing accuracy curves increase steadily, approaching 1, with rapid improvement in accuracy. The loss function curves for both the training and testing sets converge quickly, gradually decreasing towards 0, indicating no overfitting.

The experimental results show that in the CWRU dataset, inner and outer race faults are easily confused by the model, leading to classification errors. Inner race faults and rolling element faults are also frequently misclassified. However, the predictions for healthy and faulty states were entirely correct, with accurate differentiation between healthy and faulty states.

#### Experimental verification with the SEU dataset

1. Experimental setup

In this experiment, the gearbox dataset from the SEU dataset is used to validate the effectiveness of the proposed model in gearbox fault diagnosis. For this experiment, data from the operating condition of 30 Hz/1800 rpm and a load of 7.32 Nm was selected. This portion of the dataset contains five health states: normal operation, chipped tooth, missing tooth, root crack, and surface wear, with corresponding labels set as 0, 1, 2, 3, and 4.

The selected dataset is shown in Table 3. The high-frequency vibration signals were split into 1024 sub-samples, each containing 2048 data points. Each sub-sample is treated as a node in the graph, and a graph is constructed for every 8 sub-samples. The data was divided into a training set and a testing set at an 8:2 ratio for model training and testing.

**Table 3:** Gearbox data for training and testing.

Health condition	Labels	Gearboxes
Healthy	0	Health-30-2
Defect	1	Chipped-30-2
Broken teeth	2	Miss-30-2
Gear root crack	3	Root-30-2
Tooth surface wear	4	Surface-30-2

2) Experimental results and analyses

**Fig. 8:**
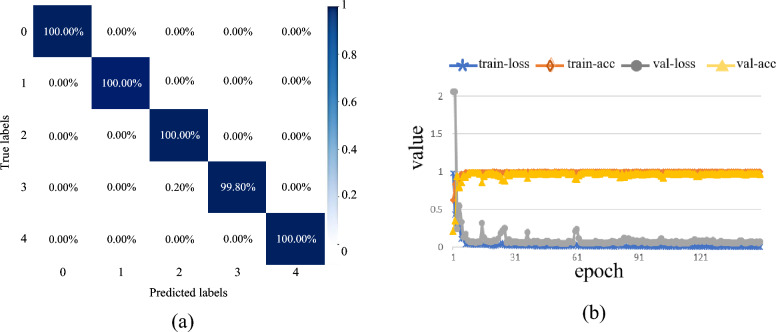
**a** Confusion matrix and **b** learning curve on SEU dataset.

The test results are shown in Fig. 8a, where the vertical coordinates of the confusion matrix represent the true fault labels of the samples and the horizontal coordinates represent the fault diagnosis results of the model. From the figure, it can be seen that 0.20% of the fault states of cracks at the root of the gears (label 3) are incorrectly predicted to be defective, while the rest of the healthy states (label 0), defective states (label 1), broken tooth states (label 2) and tooth wear states (label 4) are 100% accurately predicted.

From the model learning curves in Fig. 8b, the accuracy curves of both the training and test sets gradually converge to 1, and the accuracy improves rapidly. The loss function curve converges rapidly and tends to 0, and the model is not overfitted. The experimental results show that in the SEU gear data set, most of the fault states are accurately predicted by the model, and the cracked and defective faults at the root of the gears are easily confused, and the health state and fault state are completely and accurately predicted.

### Robustness against noise

This experiment will compare and verify the diagnostic performance of the AFFN-HGNN model under different levels of noise interference. It will also be compared with other benchmark models under the same noise conditions to verify the robustness of the model in complex environments.

1. Experimental setup

**Fig. 9:**
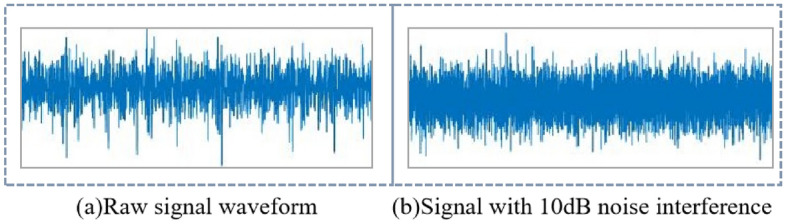
Comparison of signal waveforms under the interference of 10dB noise.

To ensure comparability of the experimental results, the model parameters and the experimental setup were remained consistent, only the noise levels were changed. The strength of the added noise was represented by the SNR (Signal-to-noise ratios). The SNR for the composite noisy signal is 0 dB, which means the power of noise is equal to that of the original signal. We test the proposed model with noisy signals ranging from –10 dB to 10 dB, i.e., -10 dB, -8 dB , -6 dB, -4 dB, -2 dB, 0 dB, 2 dB, 4 dB, 6 dB, 8 dB, 10 dB. Figure 9a shows the original vibration signal, while Fig. 9b shows the waveform after adding 10 dB of noise to the signal.

First, the diagnostic model was trained using the noise-free training data set. Then, the trained model was tested on the noisy test samples to evaluate its robustness to noise interference. The benchmark models compared in this experiment are SVM, 1D-CNN, WPT-CNN, and LSTM, respectively, using mixed-load data sampled at a frequency of 48 kHz from the CWRU dataset.

2) Experimental results and analyses

**Fig. 10:**
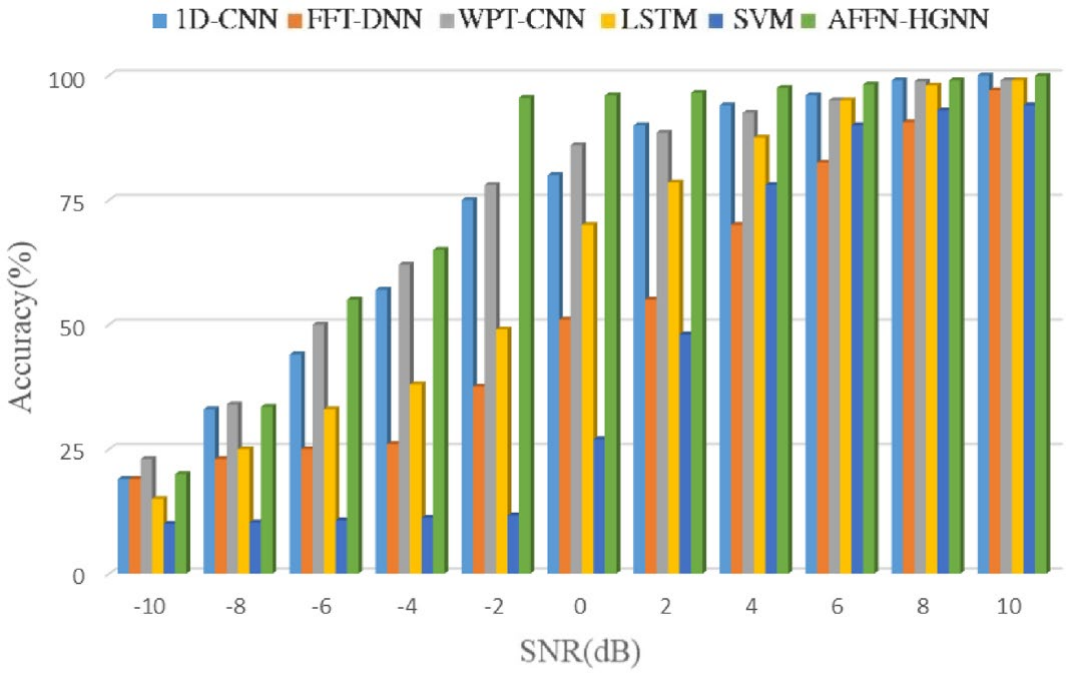
Comparison of the accuracy in different noise environments.

The fault classification accuracy on the test set is shown in Fig. 10. Compared to other algorithms, the proposed method demonstrates stronger stability under varying levels of noise interference. Specifically, when the signal-to-noise ratio (SNR) exceeds -6 dB, the performance of the AFFN-HGNN model surpasses all comparison methods. The experimental results indicate that the proposed method exhibits superior robustness in handling noise.

### Comparison with baseline methods

1. Experimental Setup

This experiment uses the Paderborn dataset to compare and validate the effectiveness of the proposed model against other fault diagnosis models. To ensure consistency in the dataset and operating conditions for the comparative experiments, the dataset is divided into four groups (A, B, C, and D) based on operating conditions. The conditions for the four datasets are shown in Table 4. For each comparative method, five repeated experiments are conducted under each operating condition, and the average of the five results is taken as the final experimental result. The datasets are split into training and testing sets in an 8:2 ratio to ensure consistency across experiments. The comparative methods include GCN^34^, MRF-GCN^35^, FDGCN^2^, Diagnosisformer^36^, and TST^37^.

- **GCN**^34^: It proposes a practical guideline and a novel, GNN-based framework for intelligent fault diagnosis and prognosis. This incorporates three graph construction strategies and various GCN and pooling architectures, which address the challenges of modelling non-Euclidean PHM data.
- **Multireceptive field graph convolutional network (MRF-GCN)**^35^: The MRF-GCN constructs weighted graphs to reflect sample relationships and uses the fusion of multiple receptive fields to extract enhanced features, thereby improving the performance of fault diagnosis in non-Euclidean spaces.
- **Fast deep graph convolutional network (FDGCN)**^2^: The FDGCN leverages wavelet packet to construct time–frequency graphs, applying graph convolution and optimised pooling to extract long-span features and enable efficient classification.
- **Diagnosisformer**^36^: A transformer-based fault diagnosis model that integrates FFT-extracted features with a multi-feature parallel fusion encoder and a cross-flipped decoder. This model achieves superior accuracy and robustness on both laboratory and CWRU bearing datasets compared to traditional deep learning methods.
- **Time Series Transformer (TST)**^37^: The TST uses a novel tokeniser to process one-dimensional vibration signals for the diagnosis of faults in rotating machinery. This enables effective feature extraction and achieves superior accuracy and class separability compared to traditional CNN and RNN models.

**Table 4:** Operating conditions of the four experimental datasets.

Datesets	Speed(rpm)	Load moment(Nm)	Radial force(N)
A	1500	0.7	1000
B	900	0.7	1000
C	1500	0.1	1000
D	1500	0.7	1000

2) Results and Analysis

**Fig. 11:**
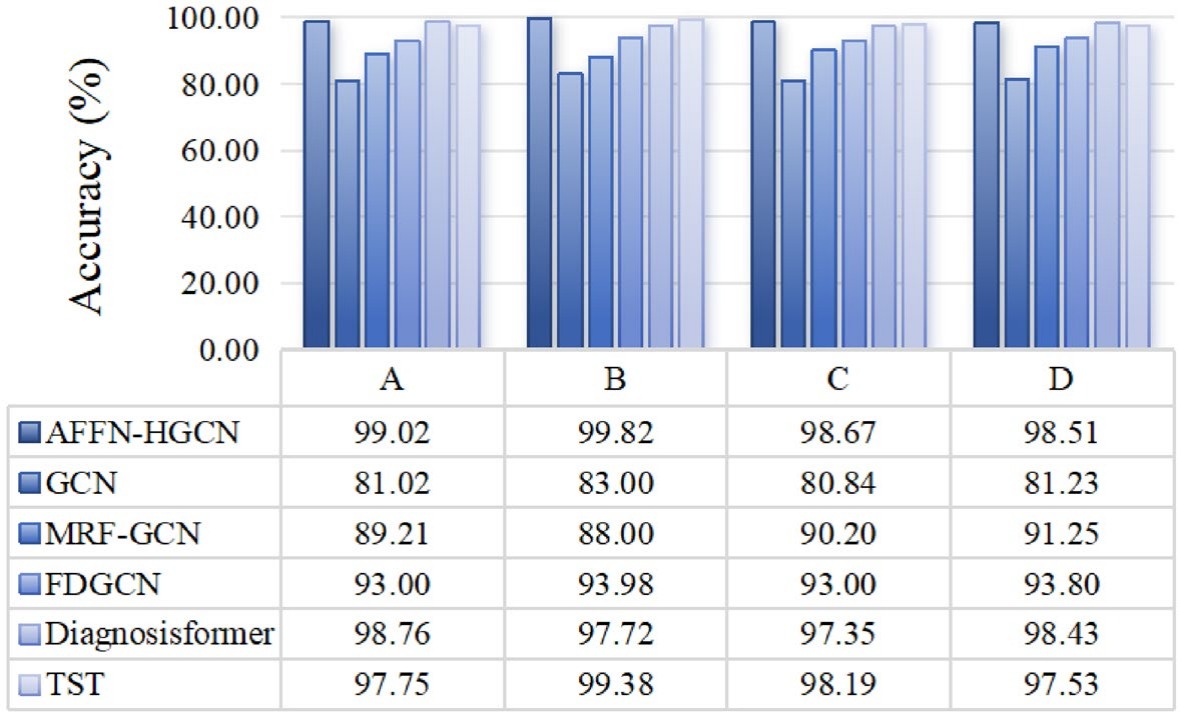
Comparison of the accuracy of different methods.

The experimental results are shown in Fig. 11. The proposed AFFN-HGNN model achieves the best classification performance, with an accuracy of over 98.5% under all four operating conditions. The traditional GCN model has a relatively lower accuracy, with its best accuracy reaching only 83% under the four conditions. This is likely due to its lack of advanced feature extraction and optimization mechanisms. The improved GCNet model achieves a best accuracy of 91.25%, while the FDGCN model reaches 93.98% under the four conditions. The Diagnosisformer and TST models show relatively higher best accuracies of 98.76% and 98.19%, respectively. These results indicate that time series analysis and signal processing still play a significant role in rotating machinery fault diagnosis. Additionally, the results further demonstrate the importance of the AFFN-HGNN algorithm’s comprehensive approach to constructing graph data.

### Ablation experiments for AFFN

1. Experimental Setup

To validate the effectiveness of the AFFN module in improving diagnostic performance, three different feature fusion methods were designed for comparative analysis. Method 1 is the AFFN-HGNN model proposed in this paper, which is based on adaptive feature fusion. Method 2 is the HGNN model with the adaptive fusion layer removed. Method 3 is the Avg-HGNN model, which employs a concatenation-based approach for feature fusion, specifically by fusing the outputs of different feature extraction layers using average weights. The experiment was conducted using real damage data from the Paderborn dataset. To ensure fairness in the results, the experimental conditions and the structure of the HGNN were kept consistent.

2) Results and Analysis

The experimental results are shown in Table 5. It can be observed that the AFFN-HGNN model correctly classifies most samples, achieving an accuracy of 98.24%, a recall of 96.15%, and an F1 score of 96.03%. Compared to the other models, the AFFN-HGNN outperforms both the HGNN and Avg-HGNN models in all metrics. The HGNN model performs the worst, indicating that removing the feature fusion layer significantly reduces its ability to capture fault features. The Avg-HGNN model performs between the AFFN-HGNN and HGNN models, suggesting that the concatenation-based fusion method establishes connections between different feature extraction layers, which improves diagnostic performance to some extent. In conclusion, the proposed adaptive fusion network effectively enhances the model’s feature learning capability and adaptability in complex graph-structured data, playing a crucial role in improving the performance of fault diagnosis models.

**Table 5:** Comparison of different fusion methods.

Evaluation metrics	Accuracy (%)	Recall (%)	F1 value (%)
AFFN-HGNN	98.24	96.15	96.03
HGNN	91.27	85.39	84.91
Avg-HGNN	96.23	92.89	92.52

### Ablation study on graph constructions

1. Experimental Setup

To quantitatively validate the contribution and complementary nature of each knowledge-based graph, a comprehensive ablation study was conducted as per the reviewer’s suggestion. The experiment was designed to evaluate the diagnostic performance of the proposed AFFN-HGNN model using the following four graph configurations on the Paderborn datasets:

- KNN only: Model using only the KNN-based graph.
- Cosine only: Model using only the cosine-similarity-based graph.
- Time only: Model using only the time-sequence-based graph.
- AFFN-HGNN: Model combining all three graphs.

All other model parameters and training settings remained identical to those described in Section 4.1 to ensure a fair comparison.

2) Results and Analysis

The results, measured by Accuracy, Precision, Recall, and F1-score, are summarized in Table 6. From the table, each single graph configuration demonstrates a certain level of diagnostic capability, confirming that each captures unique and useful information. On the Paderborn dataset, the time-sequence graph achieved the highest performance among the single-graph models, underscoring the importance of temporal dependencies for bearing fault diagnosis.

Synergistic Effect: Most importantly, the complete model, which fuses all three graphs, achieves the best performance across all metrics. This superior performance demonstrates a clear synergistic effect rather than simple aggregation. The fusion model substantially outperforms the best single-graph model by 5.79% in Accuracy, 5.05% in Recall, and 4.61% in F1-score. This significant improvement provides strong empirical evidence that the KNN, cosine similarity, and time-sequence graphs provide complementary information from local structural, global semantic, and dynamic temporal perspectives, respectively. Their integration constructs a more comprehensive and robust data representation, which is the cornerstone of our method’s effectiveness. In conclusion, this ablation study rigorously validates the necessity and effectiveness of the proposed multiple knowledge-based graph construction strategy.

**Table 6:** Ablation study on the contribution of different knowledge-based graphs.

Dataset	Graphs	Accuracy (%)	Recall (%)	F1-Score (%)
Paderborn	KNN only	85.41	83.95	84.22
	Cosine only	91.02	89.80	91.10
	Time only	92.45	91.10	91.42
	AFFN-HGNN	98.24	96.15	96.03

### Sensitivity analysis for hyperparameter $\alpha$

To evaluate the role of the hyperparameter $\alpha$ in the adaptive fusion formula, we conducted a dedicated sensitivity analysis.

**Fig. 12:**
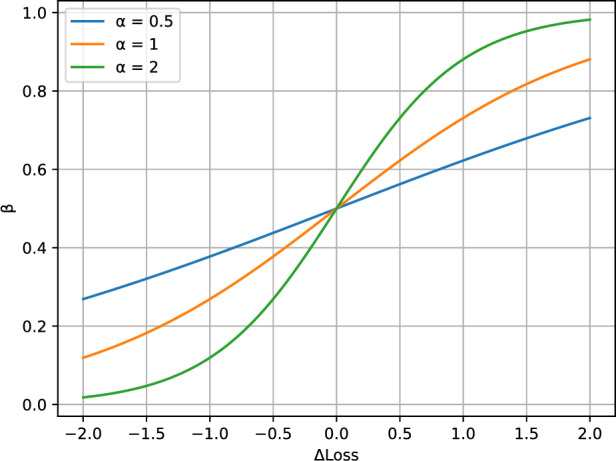
Sensitivity of $\beta$ to $\Delta Loss$ for different $\alpha$.

Theoretical Impact on Fusion Weight: The parameter $\alpha$ in the sigmoid function $\beta = 1 / (1 + \exp (-\alpha \cdot \Delta Loss))$ controls the steepness of the transition of $\beta$ from 0 to 1. This can be understood by examining its derivative with respect to $\Delta Loss$: $\frac{d\beta }{d(\Delta Loss)} = \alpha \cdot \beta \cdot (1 - \beta )$. This indicates that the maximum rate of change of $\beta$ is proportional to $\alpha$. Fig. 12 illustrates the relationship between $\Delta Loss$ and $\beta$ for different values of $\alpha$ (0.5, 1, 2). It shows that a larger $\alpha$ makes the transition sharper, causing $\beta$ to saturate more quickly to 0 or 1 for smaller absolute values of $\Delta Loss$. Conversely, a smaller $\alpha$ results in a more gradual transition.
(2) Empirical Robustness Validation: We further evaluated the empirical impact of $\alpha$ on the final diagnostic performance. Using the CWRU dataset, we trained and tested the AFFN-HGNN model with different $\alpha$ values. The results, summarized in Table 7, show that the model’s accuracy and F1-score remain stable with negligible fluctuations (within $\pm 0.5\%$) for $\alpha$ values of 0.5, 1, and 2. This demonstrates that the overall fault diagnosis performance is robust to the exact choice of $\alpha$ within a reasonable range. The adaptive fusion mechanism is effective as long as $\alpha$ is chosen to provide a meaningful response to $\Delta Loss$, and $\alpha =1$ provides a well-behaved, standard default.

**Table 7:** Model performance with different $\alpha$ values on the CWRU dataset.

$\alpha$ value	Accuracy (%)	F1-Score (%)
0.5	98.95	97.89
1.0	99.12	98.05
2.0	98.88	97.91

### Computational complexity and model efficiency analysis

To address the practical deployability of the proposed AFFN-HGNN model, we provide a comprehensive analysis of its computational complexity and efficiency, comparing it with several baseline models.

#### Theoretical complexity analysis

The computational cost of Graph Neural Networks is typically dominated by the sparse matrix operations during neighborhood aggregation. Let $|V|$ be the number of nodes, $|E|$ the number of edges, $F^{(k)}$ the input feature dimension of layer $k$, and $F^{(k+1)}$ the output feature dimension.

- GCN Layer^19^: The complexity of a single GCN layer is $O(|E|F^{(k)}F^{(k+1)} + |V|F^{(k)}F^{(k+1)})$. The first term corresponds to the edge-wise computation, and the second to the node-wise update.
- GAT Layer^20^: A single GAT layer has a complexity of $O(|E|F^{(k+1)} + |V|F^{(k)}F^{(k+1)} + |E|F^{(k)})$, which includes the cost of computing attention scores $O(|E|F^{(k)})$ and the weighted aggregation $O(|E|F^{(k+1)})$.
- AFFN-HGNN: Our model consists of one GAT layer and two GCN layers. The overall complexity is the sum of the complexities of these layers. The adaptive feature fusion network (AFFN) itself introduces negligible computational overhead, as it only involves a simple weighted sum of two feature matrices. Therefore, the overall complexity of AFFN-HGNN remains linear with respect to $|E|$ and $|V|$, i.e., O(|E| + |V|), which is efficient and scalable for large graphs.

The number of trainable parameters P in AFFN-HGNN can be calculated as follows, ignoring biases for simplicity:

$$\begin{aligned} P_{\text {GAT1}}&= F^{(0)} \times 256 \times H + 2 \times 256 \times H \quad \text {(H is the attention heads, H=6)} \\ P_{\text {GCN2}}&= 1536 \times 1536 \\ P_{\text {GCN3}}&= 1536 \times 1536 \\ P_{\text {FC1}}&= 1536 \times 512 \\ P_{\text {FC2}}&= 512 \times C \quad \text {(C is the number of fault classes)} \\ P_{\text {Total}}&\approx P_{\text {GAT1}} + P_{\text {GCN2}} + P_{\text {GCN3}} + P_{\text {FC1}} + P_{\text {FC2}} \end{aligned}$$

This parameter count is fixed for a given model architecture and does not scale with the graph size, making the model memory-efficient.

#### Discussion on practical deployability

The results lead to several key observations regarding the suitability of the GAT+GCN hybrid architecture for industrial environments:

- **Performance vs. Cost:** AFFN-HGNN achieves the highest diagnostic accuracy, outperforming all other models. This performance gain comes with an increase in computational cost (FLOPs) and model size compared to simpler models like GCN and GAT. However, this is an expected and justified trade-off.
- **Context of Cost:** Under modern computer hardware conditions, the absolute computational cost of AFFN-HGNN (32.8 million floating-point operations) is acceptable while delivering higher accuracy. This demonstrates the efficiency of graph neural network-based methods in processing structured sensor data.

## Conclusion

This paper introduces a novel Adaptive Feature Fusion Network-based Hybrid Graph Neural Networks (AFFN-HGNN) for intelligent fault diagnosis in rotating machinery. First, the model constructs multiple knowledge-based graphs to capture diverse fault characteristics from both local and global perspectives, significantly improving fault information representation. The HGNN model combines Graph Attention Networks and Graph Convolutional Networks to extract both local and global structural information. The key innovation is the adaptive feature fusion mechanism, which dynamically balances the contributions of GAT and GCN to optimize feature extraction. Powered by an adaptive learning function, the AFFN module adjusts fusion weights based on entropy and similarity losses, ensuring robust feature learning across different fault modes and noise levels.

Experimental results on three benchmark datasets (Paderborn, CWRU, and SEU) show that the proposed method achieves higher diagnostic accuracy and robustness, especially in noisy environments. Ablation studies further confirm the effectiveness of the adaptive fusion network in enhancing the model’s feature learning capabilities. Compared to traditional methods and other deep learning models, AFFN-HGNN significantly improves fault classification performance, making it a promising tool for real-time fault diagnosis in complex mechanical systems. Future work will focus on extending the model’s applicability to other machinery types and optimizing its computational efficiency for deployment on embedded systems.

## Data Availability

Data will be made available on request. Please contact the corresponding author, Gao Hui, 201716037@mail.sdu.edu.cn, for access to the data of this study.
